# Liraglutide attenuate central nervous inflammation and demyelination through AMPK and pyroptosis‐related NLRP3 pathway

**DOI:** 10.1111/cns.13791

**Published:** 2022-01-05

**Authors:** Shuang Song, Ruoyi Guo, Arshad Mehmood, Lu Zhang, Bowen Yin, Congcong Yuan, Huining Zhang, Li Guo, Bin Li

**Affiliations:** ^1^ Department of Neurology The Second Hospital of Hebei Medical University Shijiazhuang China; ^2^ Key Laboratory of Neurology of Hebei Province Shijiazhuang China; ^3^ Department of Neurology The First Hospital of Qinhuangdao Qinhuangdao China; ^4^ Department of Neurology Baoding First Central Hospital Baoding China

**Keywords:** AMPK, autophagy, experimental autoimmune encephalitis, GLP‐1R agonist, multiple sclerosis, NLPR3, pyroptosis

## Abstract

**Aims:**

Multiple sclerosis (MS) still maintains increasing prevalence and poor prognosis, while glucagon‐like peptide‐1 receptor (GLP‐1R) agonists show excellent neuroprotective capacities recently. Thus, we aim to evaluate whether the GLP‐1R agonist liraglutide (Lira) could ameliorate central nervous system demyelination and inflammation.

**Methods:**

The therapeutic effect of Lira was tested on experimental autoimmune encephalitis (EAE) in vivo and a microglia cell line BV2 in vitro.

**Results:**

Lira administration could ameliorate the disease score of EAE mice, delay the disease onset, ameliorate pathological demyelination and inflammation score in lumbar spinal cord, reduce pathogenic T helper cell transcription in spleen, restore phosphorylated adenosine monophosphate‐activated protein kinase (pAMPK) level, autophagy level, and inhibit pyroptosis‐related NLR family, pyrin domain‐containing protein 3 (NLRP3) pathway in lumbar spinal cord. Additionally, cell viability test, lactate dehydrogenase release test, and dead/live cell staining test for BV2 cells showed Lira could not salvage BV2 from nigericin‐induced pyroptosis significantly.

**Conclusion:**

Lira has anti‐inflammation and anti‐demyelination effect on EAE mice, and the protective effect of Lira in the EAE model may be related to regulation of pAMPK pathway, autophagy, and NLRP3 pathway. However, Lira treatment cannot significantly inhibit pyroptosis of BV2 cells in vitro. Our study provides Lira as a potential candidate for Multiple Sclerosis treatment.

## Graphical abstract

Liraglutide (Lira) has neuroprotective effect on experimental autoimmune encephalitis (EAE) mice, and the protective effect of Lira in the EAE model may be related to regulation of pAMPK level, autophagy, and NLRP3 pathway. However, Lira treatment cannot significantly inhibit pyroptosis of BV2 cells in vitro.

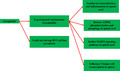

## 1 INTRODUCTION

As one of the most studied central nervous system demyelinating and autoimmune degeneration diseases, MS still maintains increasing prevalence and poor prognosis.^1^ From 2013 to 2020, the prevalence of MS has risen 14.69% to 43.95 per 100,000 individuals globally.^2^ Although many disease‐modifying therapies have been developed, new therapies are still in need. Experimental autoimmune encephalitis (EAE)^3^ is a classical animal model mimicking central nervous demyelinating lesions and T‐cell responses of MS, offering convenient chances to test potential drugs for MS.

Glucagon‐like peptide‐1 (GLP‐1) is a kind of endogenous incretin, first discovered in the early 1980s, produced from both gut and brain and exerts its role by binding to GLP‐1R, a G protein‐coupled receptor, then activating its downstream signal transduction cascades. In the digestive system, GLP‐1 is secreted from intestinal L cells by the stimulation of food intake and enhances insulin secretion from pancreatic islets, thus exerting its glucose‐dependent hypoglycemic effect, while in the nerve system, GLP‐1 is mainly secreted by preproglucagon‐expressing neurons from the nucleus tractus solitarius and can act as neurotransmitters, then regulate the activity of vagal nerve system and limbic nerve system to affect varieties of biological processes including eating behavior, weight control, emotions, energy metabolism homeostasis, cognitive abilities, and cardiovascular functions.^4^, ^5^ It has been reported that GLP‐1R exists in neurons and microglia and is widely distributed in the brain and spinal cord, especially expressed in cognitive‐related areas such as the hippocampus, giving GLP‐1 the potential for its neuroprotective effects.^6^, ^7^, ^8^, ^9^ However, endogenous GLP‐1 will be quickly inactivated by dipeptidyl peptidase‐4 (DDP‐4) after release to the extracellular space,^10^ so the pharmacological usage of which is limited.

In the 1990s, after the discovery of long half‐life DPP‐4 resistant human GLP‐1 analog exendin‐4 from the venom of Heloderma lizard, many long‐acting GLP‐1R agonists were developed based on exendin‐4 structure and approved clinically for its hypoglycemic or bodyweight lowering usage.^11^ Among them, Lira is synthesized by Novo Nordisk with Lys34Arg amino acid substitution to enhance the resistance for DDP‐4 degradation and fatty acid side chains to reduce renal filtration^12^ and was the second‐licensed GLP‐1R agonist by Food and Drug Administration (FDA) in January 2010 for type 2 diabetes treatment. Moreover, during 10 years of GLP‐1R activating therapies, the neuroprotective therapeutic efficacy of Lira and other GLP‐1R agonists has been revealed and yielded widespread attention. Not only clinical trials but also animal experiments indicate GLP‐1R agonists could improve neurodegenerative diseases, such as Parkinson's disease^13^, ^14^ and Alzheimer's disease,^15^, ^16^ while GLP‐1R also exerts therapeutic efficacy on brain ischemia,^17^, ^18^ traumatic brain injury,^19^ and psychiatric disorders^20^, ^21^, ^22^ in animal models. Besides, various kinds of DPP‐4 inhibitors, which can increase the level of endogenous GLP‐1 level, exhibit neuroprotective and cognitive protective effects either.^23^, ^24^, ^25^

GLP‐1R agonists also play a role in neuroimmune processes. In terms of MS, GLP‐1R agonists and DPP‐4 inhibitors have been reported to have therapeutic efficacy on neuroinflammation and demyelination on EAE model,^26^, ^27^ cuprizone model^28^ in vivo, and BV2 model^29^ in vitro through regulating peripheral T helper (Th) cell proportions, stabilize microglia, and reduce pro‐inflammatory cytokines. Although it is demonstrated that Lira has a therapeutic effect on the Lewi rat EAE model, delayed its disease onset, and increased nerve tissue antioxidant capacity,^30^ the effect of Lira on mice EAE model and its underlying mechanisms have not been investigated.

When GLP‐1R is activated by agonists, the G protein dissociates subsequently, activates adenylate cyclase and then generates cyclic adenosine monophosphate to trigger acute cell response. Meanwhile, activated G protein will stimulate a broad range of downstream molecules such as phosphoinositide 3‐kinase (PI3K), protein kinase B (Akt), and mitogen associated protein kinase to cause long‐term biological effects, including facilitation of insulin signaling, neurotrophy, anti‐oxidative and anti‐inflammation roles.^31^, ^32^ Recently, it has been demonstrated that Lira exerts therapeutic roles through AMPK, autophagy, and NLRP3 inflammasome in various kinds of animal or cell disease models.^33^, ^34^, ^35^, ^36^, ^37^ AMPK is a major sensor of cell metabolic status and is closely related to inflammation regulation.^38^ Phosphorylation of AMPK is related to the disease severity and treatment outcome in EAE models.^39^, ^40^ Autophagy, which is a lysosome‐dependent degradation pathway to clear potential cell toxic molecules and organelles, is in close relationship with MS pathogenesis.^41^ Impaired autophagy influx in nerve tissue may worsen EAE,^42^ and restoring autophagy levels in nerve tissue may have therapeutic effects on EAE.^43^ Similarly, NLRP3 inflammasome is a potent inflammatory signals sensor and involved in the pathogenesis of MS and EAE,^44^ which can recruit PYD and CARD domain‐containing (ASC), then activates caspase 1, then splices substrates including interleukin‐1β (IL‐1β), interleukin 18 (IL‐18), and Gasdermin D (GSDMD), and even in some situation causes pyroptosis, a newly discovered programmed cell death, which leads to cell perforation and a large amount of pro‐inflammatory cytokines release, thus may exacerbate MS/EAE.^45^

In our research, to the best of our knowledge, we tested the anti‐inflammation and anti‐demyelination effect of Lira on mice EAE model for the first time; then, we tested whether Lira intervention could salvage the microglia from pyroptosis in vitro, therefore to expand the data for pharmacological effects of Lira on demyelinating disease.

## 2 MATERIALS AND METHODS

Materials and methods can be found in Appendix S1, Tables S1 and S2.

## 3 RESULTS

### 3.1 Lira ameliorated the disease score and delayed the disease onset of EAE mice

**FIGURE 1:**
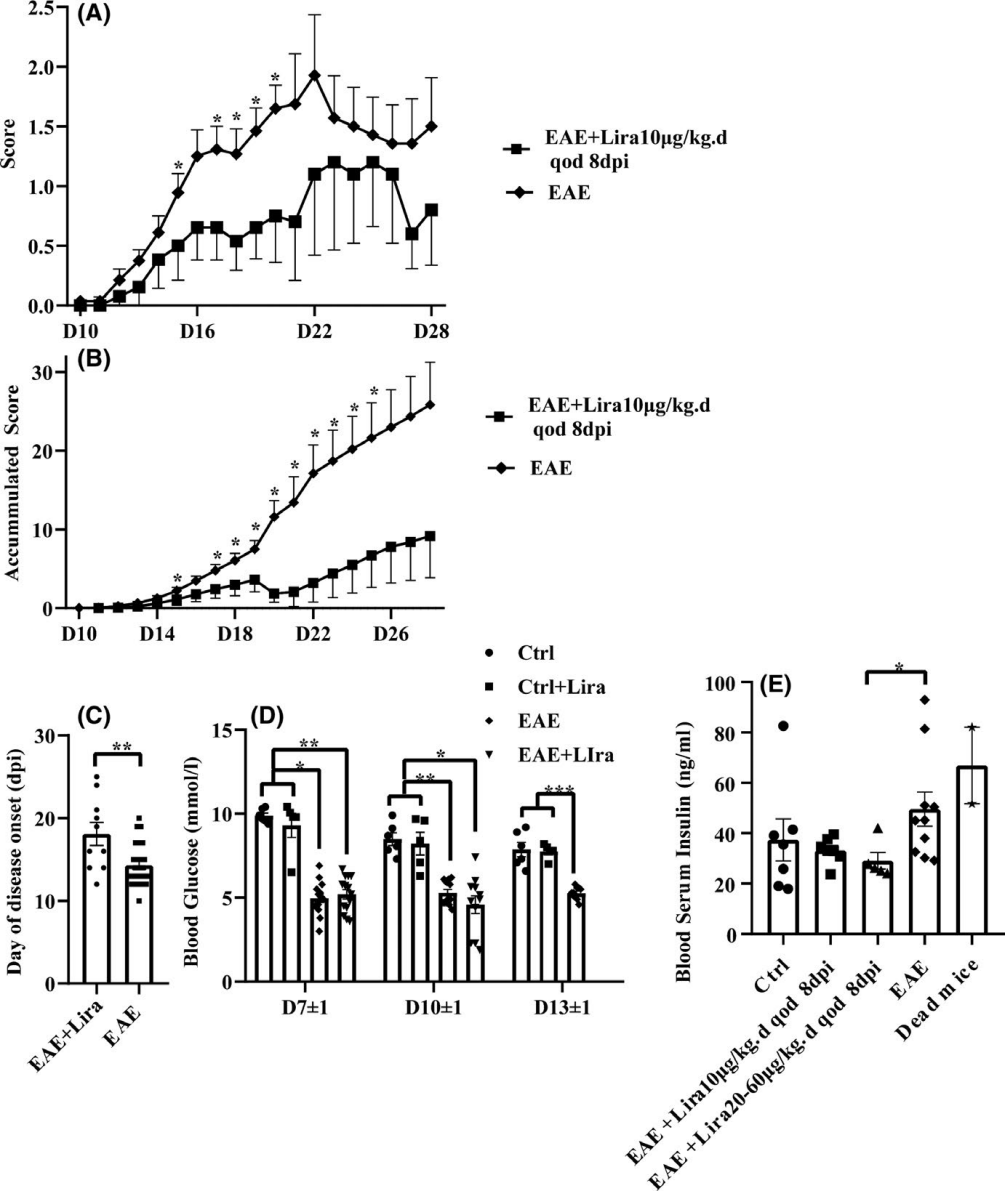
Liraglutide (Lira) administration ameliorated the disease score of experimental autoimmune encephalitis (EAE) mice and delayed the disease onset. EAE induction could reduce random blood glucose level, while Lira administration did not have an extra hypoglycemic activity. Certain dosages of Lira had insulin lowering effect compared with EAE group. Lira was administered subcutaneously (i.c.) daily (qd) or one time in 2 days (qod) after certain time points days postimmunisation (dpi). Data were shown in the form of mean ± SEM. * represents *p* < 0.05, ** represents *p* < 0.01, *** represents *p* < 0.001. (A–C) shows disease score, accumulated disease score and disease onset of EAE + Lira group and EAE group, *n* = 13–28 per group. The rating scale is 1 (paralyzed tail); 2 (posterior limb paresis); 3 (posterior limb paraplegia); 4 (posterior limb paraplegia with forelimb weakness or paralysis); 5 (moribund, or dead). Mice judged between grades received intermediate scores (±0.5). (D) shows random blood glucose level among different groups in serial time points, *n* = 5–15 per group. (E) shows random blood insulin level when mice were sacrificed, *n* = 5–10 per group. In addition, two unexpected died mice after Lira administration were tested

After 6 batches of experiments in search for the optimal and safe dosage of Lira for EAE mice, mice equivalent dosages of Lira for its clinically used human dosage (HD) exerting hyperglycemia effect were found to cause large numbers of unexpected death in EAE mice but not in healthy control (Ctrl) mice (Appendix S2, Figures [Link], [Link], Tables S3–S4). The dosage of 10 μg/kg.d (mice equivalent dosage for 1/10 of the minimum clinically used HD for its hypoglycemic activity) one time in 2 days (qod) starting from 8 days postimmunisation (dpi) was finally decided for observing its anti‐inflammation and anti‐demyelination therapeutic effect, which showed slightly improved disease score and accumulated disease score compared with EAE group after disease onset (Figure 1A,B). Moreover, disease onset was delayed by Lira administration (EAE versus EAE + Lira 14.31 ± 2.51 dpi versus 17.44 ± 4.13 dpi, *p* = 0.031) (Figure 1C).

### 3.2 EAE versus Lira in blood glucose and insulin levels

Random blood glucose was monitored every 3–4 days after immunization with the dosage of Lira as 62.5 μg/kg d (half of the minimum HD for its hypoglycemic activity) once a day (qd) after 4 dpi (Figure 1D) in batches 3 and 4. At each time point, there was no significant difference between Ctrl and Ctrl + Lira group or EAE and EAE + Lira group, suggesting that this dosage of Lira did not affect random blood glucose significantly. However, the EAE group and EAE + Lira group both had an approximate 30–50% reduction in random blood glucose level compared with the Ctrl group and Ctrl + Lira group (*p* < 0.05), suggesting that EAE induction itself could cause a decrease in random blood glucose level.

Moreover, the blood serum insulin level was monitored by enzyme‐linked immunosorbent assay (ELISA) when the animal was sacrificed or in its moribund state (Figure 1E) in batches 5 and 6, and an unsignificant increasing trend of insulin was observed in EAE group compared with Ctrl group (49.61±21.48 ng/ml versus 37.39 ± 22.06 ng/ml, *p* = 0.095). Meanwhile, the EAE + Lira group with the Lira dosage of 20–60 μg/kg d qod after 8 dpi had significantly decreased insulin level compared with the EAE group (29.11 ± 7.40 ng/ml, *p* = 0.017). Interestingly, blood serum of 2 unexpected dead mice after Lira administration was luckily obtained in their moribund state, and the values were high (82.12 ng/ml and 51.70 ng/ml, respectively).

### 3.3 Lira ameliorated demyelination as well as inflammation and regulated Th cell transcription

**FIGURE 2:**
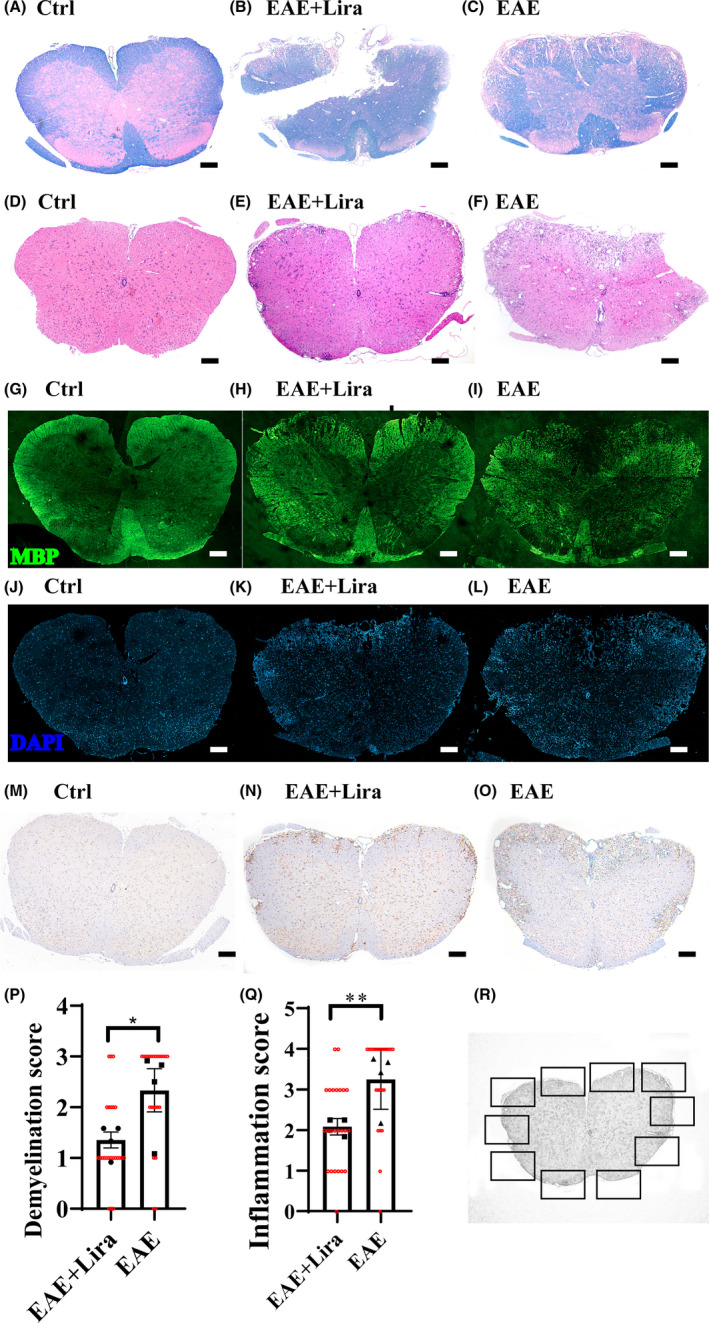
Liraglutide administration ameliorated demyelination and inflammation in lumbar spinal cord of experimental autoimmune encephalitis (EAE) mice. (A–C) shows representative lumbar spinal cord cross section Luxol fast blue (LFB) staining for different groups of mice, which reflects the degree of demyelination. (D–F) shows representative hematoxylin and eosin (HE) staining for different groups, which reflects the degree of inflammatory cell infiltration. (G–I) shows representative myelin basic protein (MBP) immunofluorescence staining for different groups. (J–L) shows representative 4′,6‐diamidino‐2‐phenylindole (DAPI) staining for different groups. (M–O) shows representative ionized calcium‐binding adapter molecule 1 (Iba‐1) immunohistochemical staining for different groups. The scale bar is 200 μm. (P and Q) shows the semi‐quantitative demyelination score (based on LFB staining) and inflammation score (based on HE staining). *N* = 4 per group. * represents *p* < 0.05, ** represents *p* < 0.01. Data were shown in the form of mean ± SEM. The black scatters represent average scores of samples, and the red scatters represent score given by the first observer. (R) Schematic figure to illustrate the process of semi‐quantitative evaluation for LFB and HE staining slices. Six of 10 fields (squares in the picture) were selected randomly by two observers blinded to the grouping information, and the average score was used to represent the extent of demyelination and inflammation for one slice. The inflammation score scale is 0 (normal); 1 (lymphocyte infiltration around meninges and blood vessels); 2 (1–10 lymphocytes in a field); 3 (11–100 lymphocytes in a field); 4 (over 100 lymphocytes in a field). And the demyelination score scale is 0 (normal); 1(small regions of sporadic myelin sheath loss); 2 (a few areas of myelin sheath loss); 3 (massive myelin sheath loss)

The protective therapeutic effect of Lira was semi‐quantitatively confirmed by Luxol fast blue (LFB) staining (Figure 2A–C) and hematoxylin and eosin (HE) staining (Figure 2D–F). The lumbar spinal cord cross sections showed massive demyelination and extensive inflammation cell infiltration foci of EAE mice on disease peak, while Ctrl group did not manifest any demyelination and inflammation signs. However, Lira administration ameliorates the demyelination and reduces the inflammation foci, with significant semi‐quantitative demyelination, and inflammation score decreased compared with the EAE group (EAE + Lira versus EAE, 1.35 ± 0.31 versus 2.33 ± 0.85 for demyelination score, and 2.08 ± 0.20 versus 3.25 ± 0.74 for inflammation score) (Figure 2P–R). In addition, representative myelin basic protein immunofluorescence staining (Figure 2G–I) also showed the same result with LFB staining findings, while representative 4′,6‐diamidino‐2‐phenylindole staining (Figure 2J–L) and ionized calcium‐binding adapter molecule 1 immunofluorescence staining (Figure 2M–O) supported the HE staining findings (statistical analysis was not conducted with these 3 morphological tests).

**FIGURE 3:**
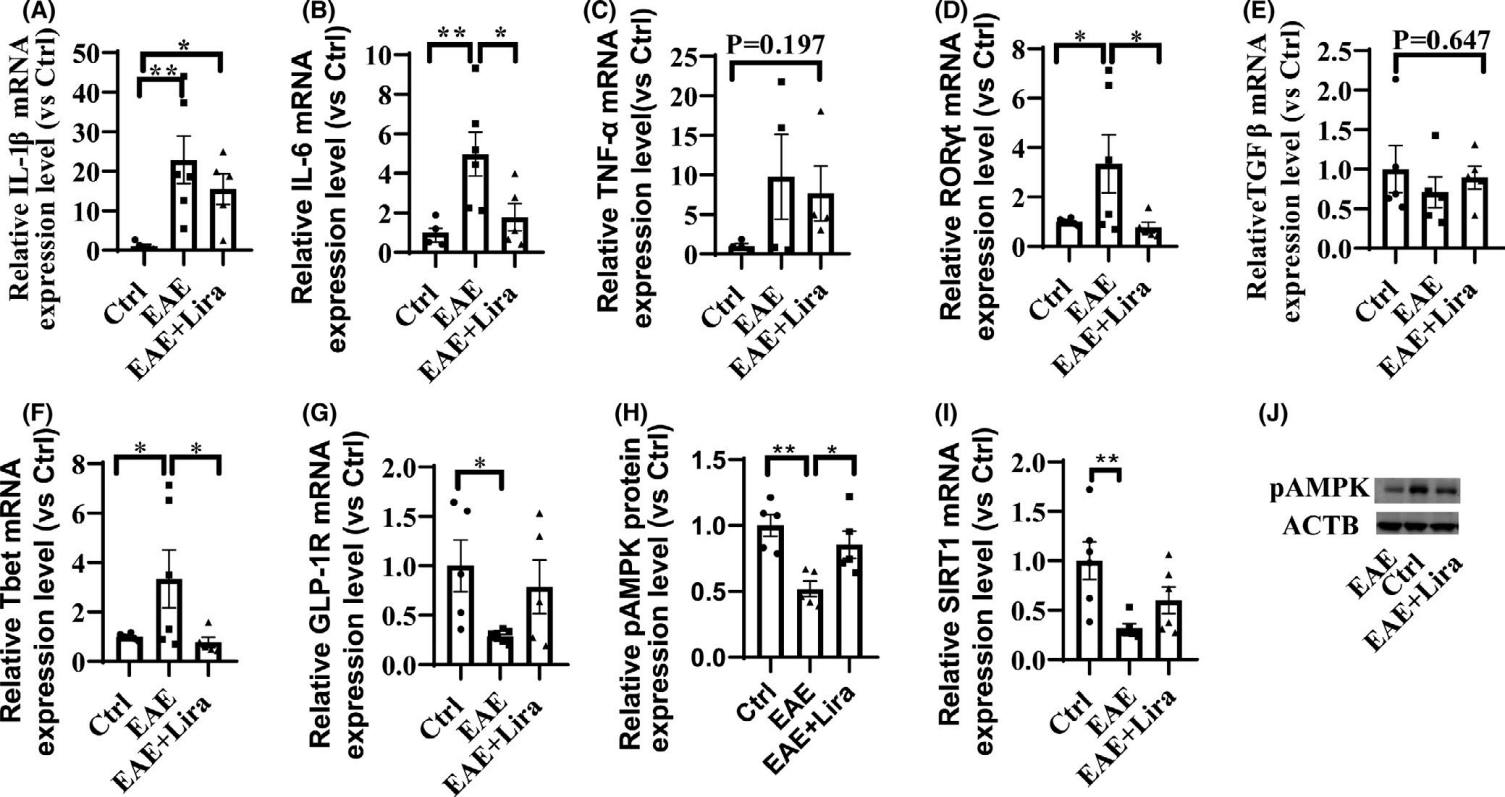
Liraglutide administration reduced pro‐inflammatory cytokines mRNA expression in nerve tissue, regulated T helper (Th) cell‐related mRNA transcription in spleen, and enhanced adenosine monophosphate‐activated protein kinase (AMPK) phosphorylation in nerve tissue (*n* ≥ 4 per group). * represents *p* < 0.05, ** represents *p* < 0.01. Data were shown in the form of mean ± SEM. (A–C) shows typical pro‐inflammatory cytokines mRNA expression level in nerve tissue. (D–F) shows typical Th cells related mRNA expression level in spleen. (G) shows GLP‐1R mRNA expression level in nerve tissue. (H and J) shows phosphorylation level of AMPK in nerve tissue. (I) shows SIRT1 mRNA expression level in nerve tissue

Moreover, Lira administration reduces key general pro‐inflammatory cytokines mRNA expression in nerve tissue (Figure 3A–C), including tumor necrosis factor‐α (TNF‐α), Interleukin‐1β (IL‐1β), and Interleukin‐6 (IL‐6). In the EAE situation, those cytokines mRNA expressions were all upregulated 5–23‐fold compared with the Ctrl group, while Lira intervention downregulated them (among them, TNF‐α did not reach statistical significance).

Th cells in the spleen are highly related to EAE pathogenesis, and Th cell‐related transcription was also found regulated by Lira intervention (Figure 3D–F). Th17 cell‐related mRNA, RAR‐related orphan receptor gamma (RORγt), and Th1 cell‐related mRNA T‐box 21 (Tbet) were upregulated by twofold in the EAE group compared with Ctrl group, which is widely considered detrimental to EAE progress, while Lira reduces these mRNA expression levels. In addition, regulatory T (Treg) (which is considered to retard the autoimmune reactions and alleviate EAE severity) cell‐related mRNA transforming growth factor‐β (TGFβ) unsiginificantly decreased in EAE situation and was restored after Lira administration.

### 3.4 The molecular protective mechanism of Lira in the EAE model

**FIGURE 4:**
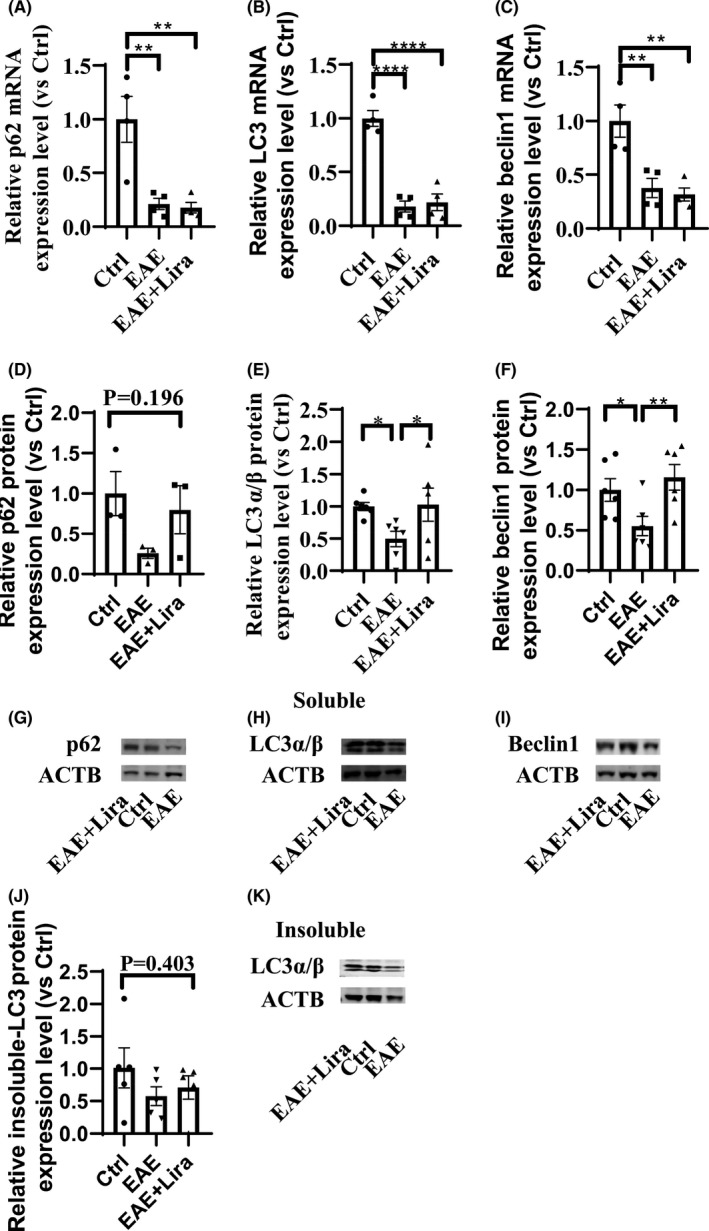
Autophagy was inhibited after experimental autoimmune encephalitis (EAE) induction, and Liraglutide intervention could partly restore the autophagy level (*n* ≥ 4 per group). * represents *p* < 0.05, ** represents *p* < 0.01, *** represents *p* < 0.001 and **** represents *p* < 0.0001. Data were shown in the form of mean ± SEM. (A–C) shows RT‐qPCR result of key autophagy indicators mRNA expression level. (D–I) shows WB result of key autophagy indicators protein expression level in soluble protein extracts. (J and K) shows WB result of LC3 protein expression level in insoluble protein extracts

Through literature review, some pathways were focused on to investigate whether they were enrolled in the protective effect of Lira on EAE. First, as it is reported that EAE could downregulate GLP‐1R expression, the GLP‐1R mRNA expression level was tested and found downregulated to 28.81 ± 5.41% of Ctrl group after EAE induction. However, Lira intervention could unsignificantly elevate the GLP‐1R expression to 78.71 ± 60.50% of the Ctrl group (Figure 3G). Next, we tested how AMPK and autophagy pathways changed after EAE induction and with Lira intervention. WB results of lumbar spinal cord exhibited significantly reduced phosphorylated AMPK expression level in EAE situation, and Lira administration partly restored the expression level (Figure 3H,J). Meanwhile, sirtuin 1 (SIRT1, downstream molecules of AMPK) mRNA expression level was found impaired in the EAE situation, and Lira intervention unsignificantly elevated it (Figure 3I). Phosphorylation of AMPK can lead to signal transduction cascades and then affect the activity of autophagy. Thus, the mRNA expression level of autophagy symbols, namely p62, LC3, and beclin1, was tested and found to have a 63–87% reduction after EAE induction, while Lira administration did not significantly affect them (Figure 4A–C). Meanwhile, p62, beclin1, and LC3 protein expression levels were also tested in EAE mice and found a significantly 37–50% decrease in soluble protein extracts (Figure 4D–I). Different from their corresponding mRNA trend, they could be restored through Lira intervention. Additionally, LC3 level in insoluble protein extracts also unsignificantly dropped in EAE and partly restored by Lira intervention (Figure 4J–K), indicating an impaired autophagy flux in EAE could be partly salvaged by Lira intervention.

**FIGURE 5:**
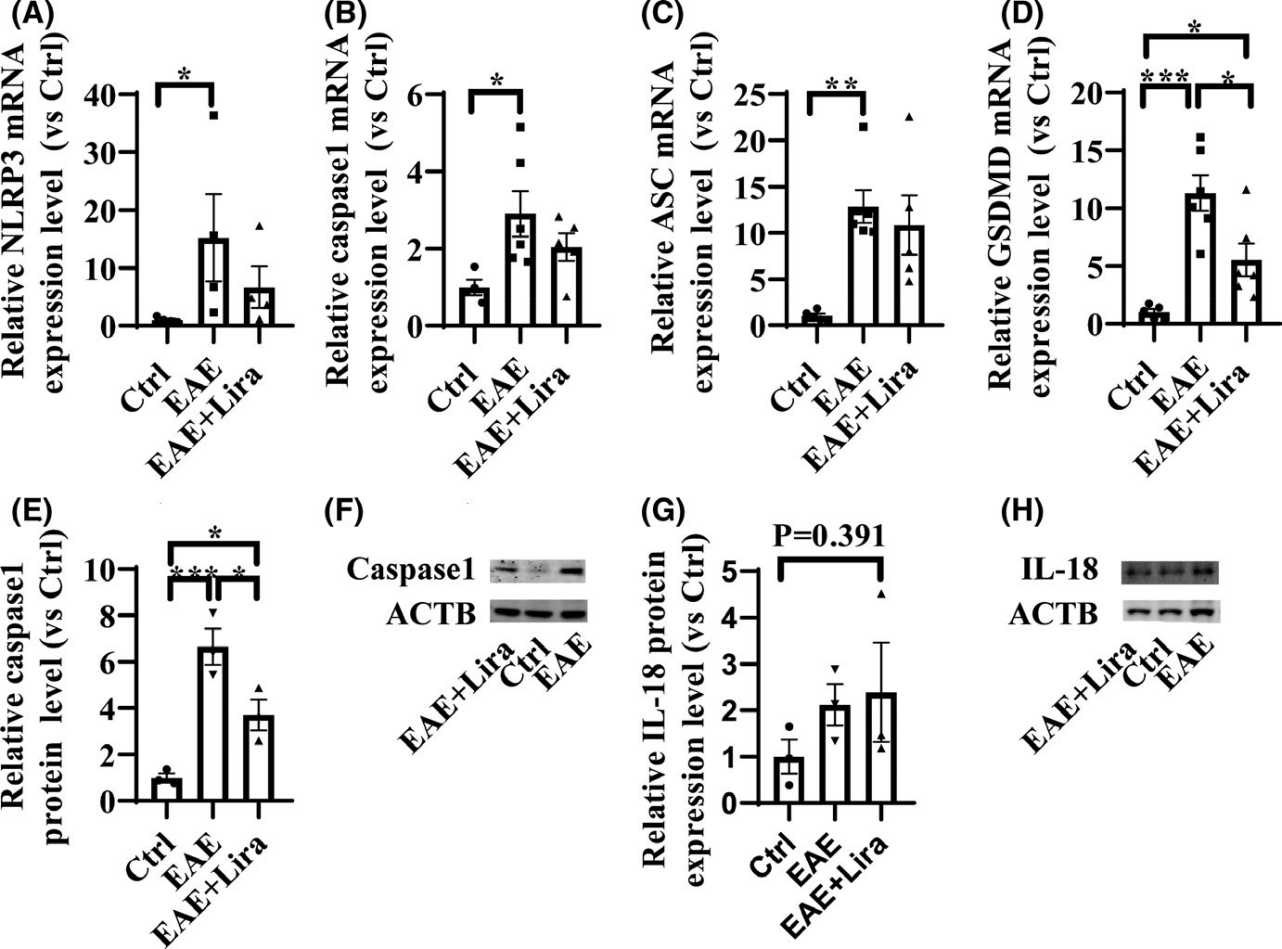
Liraglutide administration inhibited pyroptosis‐related NLR Family, pyrin domain‐containing protein 3 (NLRP3) signaling pathway (*n* ≥ 3 per group). * represents *p* < 0.05, ** represents *p* < 0.01, *** represents *p* < 0.001. Data were shown in the form of mean ± SEM. (A–D) shows result of key pyroptosis indicators mRNA expression level. (E–H) shows result of key pyroptosis indicators protein expression level

Finally, pyroptosis‐related NLRP3‐ASC‐caspase 1‐GSDMD, IL1β, and IL‐18 pathway were tested in nerve tissue. The mRNA expression level of NLRP3, ASC, caspase 1, and GSDMD increased 3–15‐fold significantly in EAE situation, while GSDMD was significantly downregulated accompanied with NLRP3 and caspase 1 unsignificantly downregulated with Lira intervention (Figure 5A–D). Similarly, caspase 1 protein expression level significantly increased, and IL‐18 protein expression level unsignificantly increased in EAE group (Figure 5E–H), but Lira intervention could only significantly downregulate the protein level of caspase 1, not IL‐18, indicating Lira intervention could partly inhibit the activated pyroptosis‐related NLRP3 pathway.

In a word, EAE could reduce the expression of GLP‐1R, downregulate the phosphorylation level of AMPK, decrease SIRT1 expression, hold back autophagy influx, and activate pyroptosis‐related NLRP3 pathway, while Lira intervention could increase phosphorylation of AMPK, elevate SIRT1 expression level, restart autophagy influx, and inhibit pyroptosis‐related NLRP3 pathway, thus may help exert its neuroprotective anti‐demyelination and anti‐inflammation roles.

### 3.5 Lira treatment in pyroptosis of BV2 cells in vitro

As the animal experiment earlier indicated, there was an inhibition effect of Lira on the NLRP3 pathway, and the previous study shows that GLP‐1R is expressed in cell membranes of microglia, a murine microglial cell line BV2 was used to construct a pyroptosis model, and test anti‐pyroptosis activity of Lira in vitro.

**FIGURE 6:**
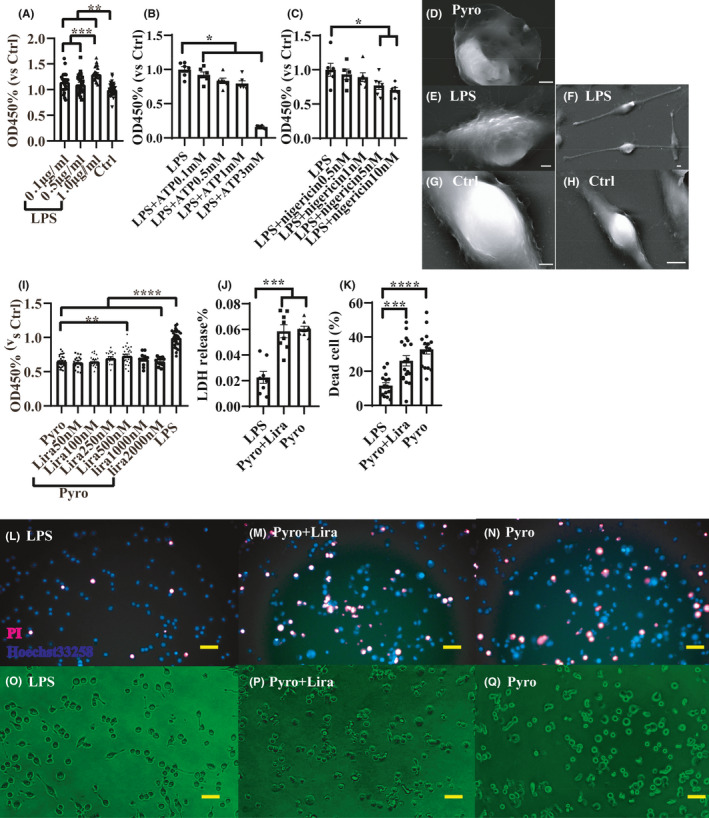
An in vitro microglia pyroptosis model showed that Liraglutide (Lira) could not significantly salvage the cell from pyroptosis. * represents *p* < 0.05, ** represents *p* < 0.01, *** represents *p* < 0.001 and **** represents *p* < 0.0001. Data were shown in the form of mean ± SEM. (A) shows Cell Counting Kit 8 (CCK8) results after 4 h of gradient concentration lipopolysaccharide (LPS) stimulation (OD450 values were compared with Ctrl group), 1.0 μg/ml LPS manifested most profound stimulation effect and was selected for subsequent experiments. Thus, in (B and C), gradient nigericin and adenosine triphosphate (ATP) were used to incubate with LPS‐primed BV2 cells for 4 h and 2 h, respectively, to induce pyroptosis, and CCK8 test was conducted (OD450 values were compared with LPS‐primed group). (D‐H) shows representative scanning electron microscope pictures of differentially treated cells. D shows a pyroptosis BV2 cell induced by nigericin, which was flattened and pored, E and F shows LPS‐primed cell, which had long projections and rugged activated soma, G and H shows a normal BV2 cell which is not primed by LPS and exhibits shorter projections and soma with smoother surface (scale bar = 5 μm). Then LPS + nigericin was chosen to build the pyroptosis model. (I) shows after incubation of gradient concentrations of Lira for 24 h then induced for pyroptosis, the CCK8 result for different groups (OD450 values were compared with LPS‐primed group). (J) shows the lactate dehydrogenase (LDH) release proportion of different groups and (K) shows dead cell proportion of different groups. (L–N) shows representative PI‐Hoechst33258 staining images of different groups and (O–Q) shows representative phase‐contrast microscope images of different groups (scale bar = 50 μm). After pyroptosis induction, BV2 cell lost projections and turns into a round shape

Through literature review, lipopolysaccharide (LPS), adenosine triphosphate (ATP), and nigericin were chosen to construct the pyroptosis model. First, a concentration gradient of LPS (4 h incubation) was tested on BV2 cells, ranging from 0.1 μg/ml to 1.0 μg/ml. Results showed that all the concentrations could activate BV2 cell and enhance its viability (Figure 6A), but 1.0 μg/ml had the statistically strongest activation effect in our gradient. Thus, 1.0 μg/ml LPS incubation for 4 h was selected for subsequent experiments. Then, LPS‐primed cells were challenged by gradient concentration of ATP (2 h) or nigericin (4 h) to induce pyroptosis. Results showed that ATP concentration above 0.1 mM and nigericin concentration above 5 nM showed the significant effect to dampen the cell viability (Figure 6B,C), and under the phase‐contrast microscopy, cells manifested round shape and lost their projections after the challenge (Figure 6O–Q). Furthermore, with the validation of SEM morphological evaluation, the cells challenged by nigericin (Figure 6D) showed a flattened shape with pores on the cell body, which was characterized for pyroptosis, while LPS‐primed cells (Figure 6E,F) showed extended projections and coarse surface of soma compared with unprimed negative cell (Figure 6G,H), indicating its activated state. However, a large proportion of ATP‐challenged cells showed apoptosis‐like characters such as cell shrinkage and membrane blebbing (data not shown); thus, nigericin was chosen for subsequent experiments.

Next, gradient concentrations of Lira ranging from 50 nM to 2000 nM were used to incubate the BV2 cell for 24 h as a preventive treatment, while in the LPS group and pyroptosis (Pyro) group, the cell was incubated with Dulbecco's Modified Eagle Medium (DMEM) instead. After the incubation, cells were induced to pyroptosis except for the LPS group (in the LPS group, cells were only primed by LPS, not challenged by nigericin), and then, CCK8 test was performed and the value of OD450 in every group was compared with the LPS group for normalization. Results (Figure 6I) showed that only the concentration of 500 nM Lira had a significant protective effect on pyroptosis. However, the experiments on this concentration were repeated 5 times, and only 2 times gave positive results, indicating Lira was not strong enough to stop pyroptosis. Still, the Lira concentration of 500 nM was chosen for the subsequent experiment.

Furthermore, lactate dehydrogenase (LDH) release experiment and dead/live cell staining experiment were conducted for further validation of anti‐pyroptosis effect of Lira, among LPS group (only primed by LPS not challenged by nigericin after 24 h incubation with DMEM), Pyro group (induced for pyroptosis after 24 h incubation with DMEM), and Pyro + Lira group (induced for pyroptosis after incubation with 500 nM Lira for 24 h). Results showed that the Pyro group had 3 times increase in LDH release compared with the LPS group (Figure 6J), while there was no statistical difference between the Pyro group and Pyro + Lira group. Similarly, dead cell proportion increased 3 times in the Pyro group, but it was not statistically different from Pyro + Lira group (Figure 6K,L–Q).

To sum up, Lira could not significantly stop the pyroptosis of BV2 cells in vitro but had a trend to ameliorate it.

## 4 DISCUSSION

In this study, Lira, to our knowledge, for the first time to be demonstrated having anti‐inflammation and anti‐demyelination roles in the mice EAE model. Similar to the effect of Lira on the rat EAE model,^30^ Lira significantly delays the disease onset and alleviates the disease severity. Moreover, as a supplement to a previous study on rats, the neuroprotective effect was also semi‐quantitatively confirmed pathologically, with less inflammatory cell infiltration and white matter demyelination in EAE model.

GLP‐1R is distributed in immune organs, including thymus and spleen, and various types of immune cells such as T cells,^46^, ^47^ natural killer T cells,^48^ microglia,^49^ monocytes, and macrophages,^50^ being capable of regulating peripheral Treg cells proportion.^46^ Moreover, in previous research, Th17 and Th1 infiltration in nerve tissue are reduced by GLP‐1R activation in the EAE model.^26^ In line with this finding, Lira regulated the Th cell‐related transcription in the spleen, reduced the pathogenic Th1‐and Th17‐related transcriptions in our data. GLP‐1R is observed to accumulate after nerve tissue injury,^49^, ^51^ but decreased in EAE‐challenged nerve tissue^27^, ^52^ and LPS‐primed microglia,^27^ indicating impaired GLP‐1R downstream pathways may participate in the pathogenesis of EAE. In our study, GLP‐1R also decreased during the acute phase of EAE and unsignificantly restored by GLP‐1R activation. Lira is demonstrated to ameliorate neuroinflammation, improve memory function, and reduce amyloid‐β deposition and oxidative injury in Alzheimer's disease animal model,^53^, ^54^, ^55^ and the mechanisms include but not limited to facilitating insulin signaling pathways, activating cAMP/PKA pathways, and PI3K/Akt pathways, thus restored GLP‐1R level might benefit to exert neuroprotective roles.

AMPK can downregulate inflammation pathways through many downstream molecules such as SIRT1, p53, and peroxisome proliferator‐activated receptor γ coactivator‐1, then inhibit nuclear factor‐kB, and indirectly suppress pro‐inflammatory gene transcriptions.^56^, ^57^ Moreover, activation of AMPK can phosphorylate tuberous sclerosis complex 1/tuberous sclerosis complex 2 (TSC1/TSC2) and raptor, affect mechanistic target of rapamycin complex 1 (mTORC1) and then promote autophagy level.^58^ There was a restoration of pAMPK and its downstream SIRT1 expression level after Lira treatment in our data, but previous findings of the interactions between Lira and AMPK are controversial. On the one hand, accumulating evidence showed that GLP‐1R activation could elevate pAMPK and autophagy level,^34^, ^59^ but on the other hand, Lira can mainly activate PI3K/Akt pathways through GLP‐1R signal transduction, which may elevate cell ATP level, inhibit downstream TSC1/TSC2, or directly phosphorylate AMPK at Ser^485^ in α1 subunit or Ser^491^ in α2 subunit, to reduce Thr^172^ phosphorylation then inhibit AMPK activity and autophagy.^60^, ^61^, ^62^ The effect and mechanism of GLP‐1R activation on AMPK phosphorylation should be investigated in more details in the future. LC3, p62, and beclin1 are the main molecules facilitating autophagosome assembly and representing autophagy level, while there are also ambivalent findings of whether these autophagy indicators are upregulated or downregulated in the spinal cord after EAE induction,^42^, ^43^, ^63^ probably because that spinal cord contains a plethora of different cells, and autophagy might play different roles in different types of cells in EAE pathogenesis, including positively contributing to both autoimmune T‐cell pathogenicity and neuron survival, thus yielding variable results.^41^ In our study, the overall spinal cord level of beclin1, p62, and LC3 was all downregulated after EAE induction, indicating an impaired autophagy influx within the spinal cord, while Lira could partly restore it.

Accumulating evidence showed that NLRP3 inflammasome is activated during the pathogenesis of MS/EAE, and its downstream products, including IL‐1β and IL‐18, can compromise the blood‐brain barrier, induce neural toxicity, stimulate autoimmune T cells and then deteriorate MS/EAE.^44^, ^45^, ^64^, ^65^ Moreover, NLRP3 can activate caspase 1 and splice GSDMD, then form membranes pore to induce pyroptosis, and exacerbate the inflammation. In our study, the overall level of pyroptosis‐related NLRP3 pathway indicators was upregulated after EAE induction, in line with previous data, while partly restored by Lira intervention, suggesting an anti‐pyroptosis effect of Lira. Actually, Lira has already been demonstrated to ameliorate pyroptosis in cardiomyoblast cell lines (H9c2) and hepatocellular carcinoma (HepG2) cell lines through increasing SIRT1 expression level, reducing intracellular reactive oxygen species, and promoting mitophagy.^33^, ^35^ Microglia is a kind of important residential innate immune cell, participates in demyelinating pathogenesis, and incretins showed properties to alleviate microglia activation, reduce oxidative stress, and pro‐inflammatory cytokines transcription.^29^, ^66^, ^67^ In our study, we tested whether Lira can significantly alleviate the pyroptosis of microglia and receive a negative result. However, there was still an alleviation trend, and we only tested on cell lines; thus, experiments of Lira on primary microglia culture can be done to validate the result.

Interestingly, the dosage of Lira we tested on EAE is only 1/10 of the minimum HD for its hypoglycemic effect. According to our calculation, Lira was tested safe for its threefold minimum hypoglycemic HD on rat EAE model,^30^ and another GLP‐1R agonist dulaglutide was tested safe for its minimum hypoglycemic HD on mice EAE model.^26^ On the one hand, our results implicated that Lira may exert its anti‐inflammatory and anti‐demyelinating effects independent of its hypoglycemic effect; on the other hand, possible reasons for unexpected death for EAE mice after Lira administration remain to be investigated. A possible conjecture we made is the EAE mice would lose weight, and Lira administration might cause extra body weight loss and probably some digestive system symptoms such as nausea and anorexia, then cause general weakness and possible hypoglycemia, and finally cause unexpected death. In this regard, we monitored the random blood glucose level and insulin level accordingly. Intriguingly, EAE itself caused significant blood glucose reduction, but Lira did not have an extra hypoglycemic effect. Moreover, there was an unsignificant upregulating trend of blood serum insulin level after EAE induction. Through literature review, we do not acquire much information about the relationship between EAE and insulin excretion, which should be investigated more in the future.

## 5 LIMITATIONS AND FUTURE PERSPECTIVES

The reason for unexpected death caused by Lira administration in the mice EAE model could be investigated, and serial dosage of Lira as either prophylactic or therapeutic interventions could be tried on the EAE model to find the optimal dosage. Moreover, the mechanism of the neuroprotective effect of Lira should be investigated in‐depth in the future.

## 6 CONCLUSION

Liraglutide administration could ameliorate the disease score of EAE mice and delay the disease onset, ameliorate demyelination and inflammation in nerve tissue, and regulate Th cell transcription in the spleen of EAE mice. The protective effect of liraglutide in the EAE model may be related to regulation of AMPK pathway and autophagy, as well as inhibition of pyroptosis‐related NLRP3 pathway, but liraglutide treatment could not significantly inhibit pyroptosis of BV2 cells in vitro. Our study provides liraglutide as a potential therapeutic candidate for MS treatment.

## CONFLICT OF INTEREST

The authors declare that they have no competing interests.

## AUTHOR CONTRIBUTIONS

All the authors have accepted responsibility for the entire content of this submitted manuscript and approved submission.

## CONSENT TO PARTICIPATE

This article does not contain any studies with human participants performed by any of the authors.

## Supporting information

Fig S1

Fig S2

App S1

Tab S1‐S2

App S2

## Data Availability

The supplementary material for this article can be found online. All processed data used in this study can be obtained from the corresponding author on reasonable request.
