# Supplementary material for: Liraglutide attenuate central nervous inflammation and demyelination through AMPK and pyroptosis‐related NLRP3 pathway
Source: CNS Neurosci Ther. 2022 Jan 5;28(3):422–34. doi: 10.1111/cns.13791 (PMC8841291; doi:10.1111/cns.13791)
Supplement: Supplementary file 1 — Fig S1 [file CNS-28-422-s001.doc]

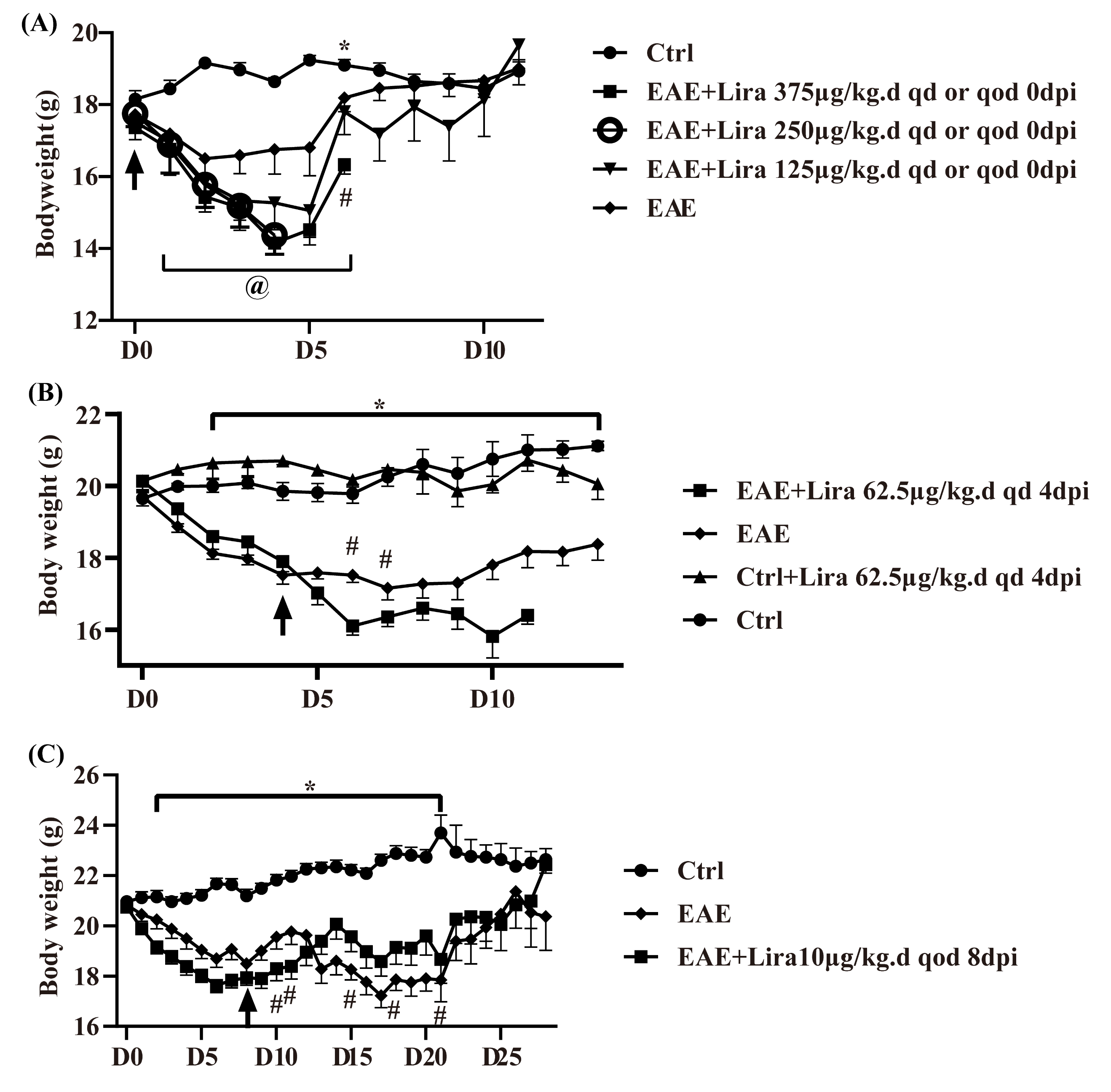


**FIGURE S1** The body weight change of mice in different batches showed that experimental autoimmune encephalitis (EAE) could induce a significant body weight loss after immunisation, and certain dosages of liraglutide (Lira) could induce significant extra body weight loss. Data were shown in the form of mean±SEM. The black arrow shows the timepoint when treatment groups started to receive Lira injections. Lira was administered subcutaneously (i.c.) daily (qd) or one time two days (qod) after certain timepoints days post immunisation (dpi). (A) Illustration of the mice body weight in batch 1. The 375μg/kg.d, 250μg/kg.d and 125μg/kg.d were selected to represent high-dose, medium-dose and low-dose of Lira administration equivalent to human hypoglycemic dosage. Lira was administered i.c. qd or qod after 0 dpi. * represents EAE versus Ctrl *p* < 0.05, ^#^ represents high-dose Lira versus EAE *p* < 0.05, @ represents *p* value of multigroup test < 0.05. (B) Illustration of the mice body weight in batch 3 and 4. The dosage 62.5 μg/kg.d Lira i.c. qd starting from 4 dpi was selected in these batches. * represents EAE versus Ctrl *p* < 0.05, ^#^ represents EAE+Lira versus EAE *p* < 0.05. (C) Illustration of the mice body weight change of different treatment groups in batch 5. * represents EAE versus Ctrl *p* < 0.05, ^#^ represents EAE+Lira versus EAE *p* < 0.05.
