# Supplementary material for: Liraglutide attenuate central nervous inflammation and demyelination through AMPK and pyroptosis‐related NLRP3 pathway
Source: CNS Neurosci Ther. 2022 Jan 5;28(3):422–34. doi: 10.1111/cns.13791 (PMC8841291; doi:10.1111/cns.13791)
Supplement: Supplementary file 2 — Fig S2 [file CNS-28-422-s004.doc]

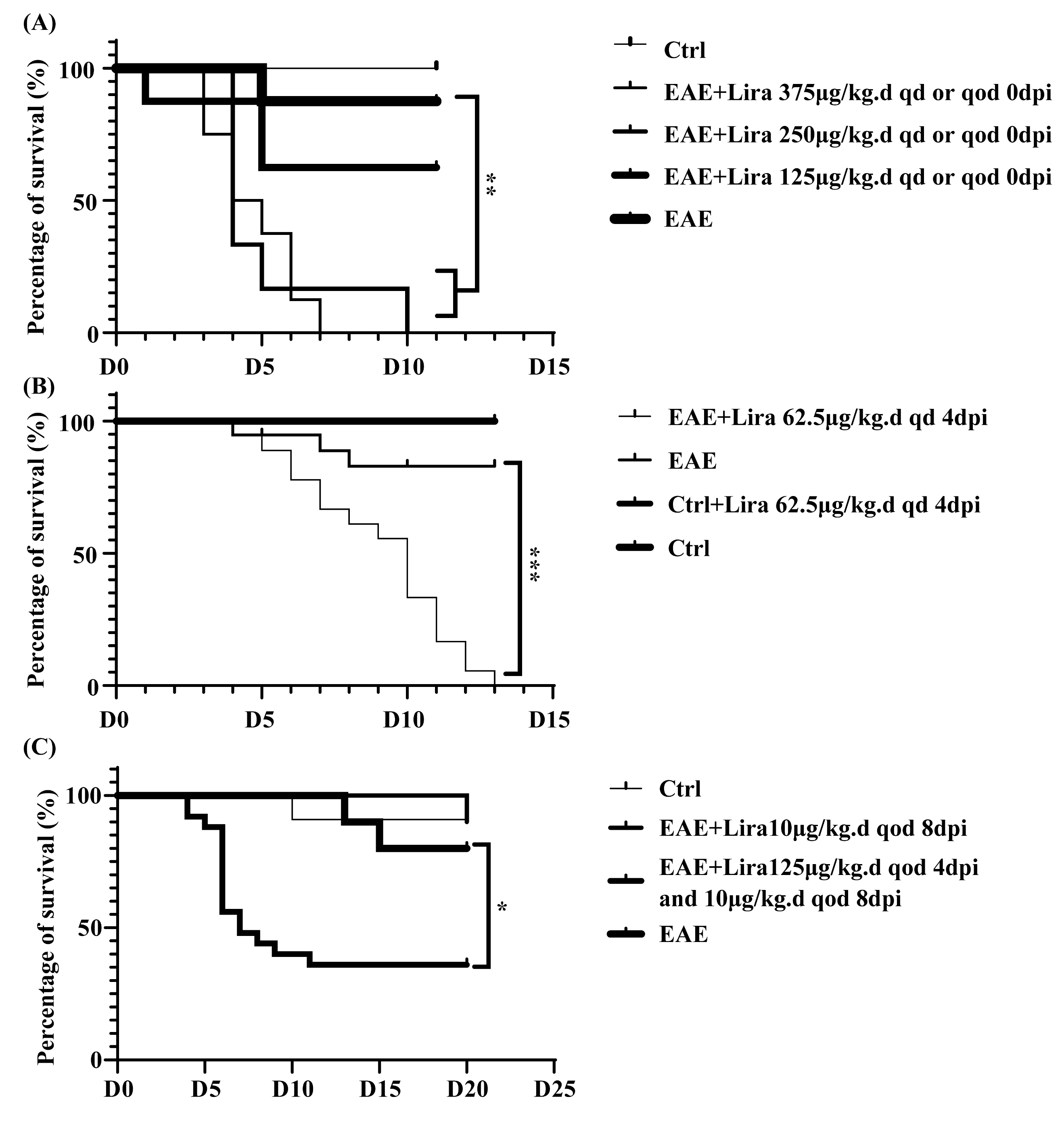


**FIGURE S2**  Kaplan-Meier curve shows that Liraglutide administration in experimental autoimmune encephalitis mice caused unexpected death. (A) Illustration of the Kaplan-Meier curve in batch 1, ** represents p < 0.01. (B) Illustration of the Kaplan-Meier curve in batch 3 and 4. *** represents p < 0.001. (C) Illustration of the Kaplan-Meier curve in batch 5. * represents p < 0.05.
