# Supplementary material for: Liraglutide attenuate central nervous inflammation and demyelination through AMPK and pyroptosis‐related NLRP3 pathway
Source: CNS Neurosci Ther. 2022 Jan 5;28(3):422–34. doi: 10.1111/cns.13791 (PMC8841291; doi:10.1111/cns.13791)
Supplement: Supplementary file 3 — App S1 [file CNS-28-422-s005.docx]

**Appendix S1**

**MATERIALS AND METHODS**

The experiments were performed at Key Laboratory of Neurology of Hebei Province, City Shijiazhuang, Province Hebei, China, which was approved by the Experimental Ethics Committee of the Second Hospital of Hebei Medical University (No.2021-AE043).

**Experimental animals and drug administration**

Female C57BL/6 mice of 8 to 10 weeks old were selected (see Table S1 for detail). Mice were allowed a week for environment adaption (24±2°C with 12 hours light-dark diurnal cycle, and food ad libitum). Then, animals were stratified according to weight and grouped using the random number table method.

Lira was purchased at a local pharmacy and diluted by sterilized saline. Different dosages of Lira for mice were converted from clinically used human dosage according to Food and Drug Administration (FDA) recommended equations^1^. Briefly, the principal equation was: human equivalent dose = animal dose in mg/kg × (animal weight in kg/human weight in kg)^0.33^. Adult human body weight was assumed to be 60 kg. Thus, to convert human dose to animal equivalent dosage, using the following convenient equation: mice dose in mg/kg = human dose in mg/kg ÷ 0.08.

The drug was administered to mice subcutaneously (i.c.) once a day (qd) or one time two days (qod) after different timepoints in experiments.

**Animal modeling and tissue preparation**

**Experimental autoimmune encephalitis induction**

EAE was induced by 4 points paraspinal subcutaneous injection with the emulsion containing 200-250 μg MOG35-55 peptide (MEVGWYRSPFSRVVHLYRNGK, Nanjing Peptide Biotech Ltd., Nanjing, China), 0.05 ml Complete Freund’s Adjuvant (Sigma, StLouis, USA), 0.05 ml sterilized saline and 4 mg/ml Mycobacterium tuberculosis H37Ra (Difco Laboratories, Detroit, USA), followed by intraperitoneal injection of 250-200 ng pertussis toxin (List Biological, CA, USA) at the time of immunization and again after 48 hours. After immunization, the body weight of mice was monitored daily.

**Animal sacrifice and tissue preparation**

Mice were sacrificed at disease peak by neck-breaking, while 3-5 mice of every group were reserved to 28 days post immunisation for whole disease course observation. For molecular biological experiments, including the western blot (WB) test and reverse transcriptive quantitative polymerase chain reaction (RT-qPCR) test, target tissues were isolated carefully and quickly on the ice, then flash-frozen with liquid nitrogen and stored in -80°C refrigerators. For enzyme-linked immunosorbent assay (ELISA), the animal blood samples were collected by removing the eyes. For morphological tests of EAE model, the freshly obtained lumbar spinal cord was quickly sectioned and immersed in 4% PFA at room temperature for more than 24 hours.

**Disease severity evaluation**

**Disease score rating**

The disease score of the EAE model was semi-quantitatively evaluated blindly and daily after immunization using a five-point scoring scale as previously reported ^2^. The accumulated score was defined as the sum of daily disease scores before the calculated day.

**Pathological staining and evaluation**

**Paraformaldehyde-fixed tissue processing and section**

PFA fixed tissues were appropriately trimmed and dehydrated by the serial concentration of ethanol, transparent in xylene, and embedded in paraffin, then sectioned serially by a microtome (5μm). A cross-section of the lumbar spinal cord was selected. Before staining, the sections were rehydrated by xylene and serial ethanol. Moreover, the sections were repaired in sodium citrate buffer (pH = 6.0) for immunohistochemical (IHC) or immunofluorescence (IF) staining.

**Hematoxylin-eosin staining and Luxol fast blue staining**

Hematoxylin-eosin (HE) staining and Luxol fast blue (LFB) staining were conducted with staining kits (G1030, G1001, G1004, Servicebio, Wuhan, China) following the manufacturer's instructions. When LFB staining was done, slices were counterstained by eosin.

**Immunohistochemical staining**

IHC staining was conducted with a staining kit (SP-9001, Zhongshan Goldenbridge, Beijing, China), and a DAB kit (G1212, Servicebio, Wuhan, China) was used for final color development following the manufacturer's instructions. Slices were counterstained by hematoxylin to show the cell nucleus.

**Immunofluorescence staining**

Slices were treated with blocking buffer (P0260, Beyotime, Shanghai, China), then incubated with the first antibody overnight at 4℃, and with fluorochrome-conjugated secondary antibody for 1 hour at room temperature, then counterstained by 4',6-diamidino-2-phenylindole (DAPI, G1012, Servicebio, Wuhan, China) to show cell nucleus. Between every two steps, slices were washed by phosphate buffer saline 3 times. Images were filmed under an Olympus FV1000 confocal fluorescence microscope. The antibodies used were listed inTable S1.

**Pathological demyelination and inflammation evaluation**

Demyelination and inflammation were semi-quantitatively evaluated using previous scoring criteria ^2^.

**Enzyme-linked immunosorbent assay**

Mice blood serum insulin level was determined by a mouse insulin competitive ELISA kit (EK2220, Multisciences, Hangzhou, China) following the manufacturer's instructions.

**Western blot analysis**

Samples were mechanically dissolved on ice in lysis buffer (P0013, Beyotime, Shanghai, China) with a protease inhibitor cocktail (P1050, Beyotime, Shanghai, China). After centrifuging at 12000 × g for 5 minutes, the supernatant of the sample was defined as soluble protein. Meanwhile, the precipitation of sample was washed by 2% sodium dodecyl sulfate solution supplemented with 1mM phenylmethanesulfonyl fluoride twice and dissolved in lysis buffer supplemented with protease inhibitor cocktail as described above, placed in a 60°C water bath for 1 hour, centrifuged at 12000 × g for 5 minutes, and then the supernatant was defined as insoluble protein. Subsequently, a protein quantitative kit (G2026, Servicebio, Wuhan, China) was used to determine the protein concentration. For denaturation, protein samples were boiled with loading buffer for 5 minutes. Next, every 20-40μg protein was loaded onto gel lanes, resolved by 8-12% sodium dodecyl sulfate-polyacrylamide gel electrophoresis according to their molecular weights, and then transferred onto polyvinylidene difluoride membranes (Millipore, Billerica, USA). The membranes were then blocked by block buffer (P0252, Beyotime, Shanghai, China) for 15 minutes and incubated with first antibodies overnight at 4℃. Subsequently, membranes were incubated by fluorochrome-conjugated secondary antibody and then developed on a far-infrared laser scanning system (LI-COR, Lincoln, USA), and the image of the WB result was analyzed by ImageJ. The relative expression levels of interested protein were adjusted according to the internal reference protein. The antibodies used in WB analysis were displayed in Table S1.

**RT-qPCR analysis**

Total RNA was extracted by TRI RNA reagent (ZS-M11008, Supersmart, Tianjin, China), isolated by chloroform, precipitated by isopropanol, washed by ethanol, dissolved in RNAase-free water, measured by absorbance at 260 nm and 280 nm, and reverse transcribed into complementary deoxyribonucleic acid (cDNA) library using SweScript RT II First Strand cDNA Synthesis Kit (G3332, Servicebio, Wuhan, China), then interested mRNA expression level was determined by RT-qPCR using 2×SYBR Green qPCR Master Mix (G3322, Servicebio, Wuhan, China). The relative expression levels of interested mRNA were normalized to β-actin (ACTB) mRNA expression level and then compared with each other using the cycle threshold (CT) method (2^-ΔΔCT^ as sample’s value). The primers are listed in Table S2.

**Random blood glucose test**

Blood glucose levels were measured by a human glucose meter (Abbott, Fermoy, Ireland) in venous blood obtained from the tail. Before the test, mice did not undergo fasting, but the test time was between 8 a.m. to 10 a.m.

**BV2 cell line experiment**

**Cell culturing**

A mouse microglial cell line BV2 (Procell, Wuhan, China) was cultured in high glucose Dulbecco's Modified Eagle Medium (DMEM, G4510, Servicebio, Wuhan, China) in a 37°C incubator filled with 5% CO_2_. The culture medium was supplemented with 1% Penicillin-Streptomycin Liquid (P1400, Solarbio, Shanghai, China) as well as 10% fetal bovine serum (AusgeneX, Molendinar, Australia). The morphology of cells was monitored under a phase-contrast microscope. The culture medium was refreshed 3 times a week, and when the cell reached 80-90% confluency, they would be passaged to new plates.

**Cell counting kit 8 test**

Cell counting kit 8 (CCK8) test was conducted to evaluate the cell viability among different treatment groups. First, 10^5^ cells/well were seeded into a 96-well plate and allowed to grow until they reached 50% confluency. Then after treatment, 10% (v/v) CCK-8 was added into the medium for 2 hours, and absorbance at 450nm was measured by a microplate reader.

**Dead/live cell staining with propidium iodide and** **Hoechst33258**

Cells were seeded into a 96-well plate, given different treatments, and then stained by propidium iodide (PI) and Hoechst33258 (represent dead and live-cell, respectively) following manufacturer's instructions (C0080 and C0021, Solarbio, Beijing, China). Both fluorescent and bright-field images were obtained by an inverted fluorescent microscope. The dead and live cells in different pictures were automatically counted by ImageJ software, and then the dead cell proportion was calculated.

**Lactate dehydrogenase release test**

10^5^ cells/well were seeded into a 96-well plate, and when they had reached 50% confluency, various treatments were given to them, then the lactate dehydrogenase (LDH) level in the supernatants of BV2 culture was measured by LDH Cytotoxicity Assay Kit (C0016 Beyotime, Shanghai, China) according to its instructions. Absorbance at 490nm and 600nm for every sample was determined by a microplate reader. The LDH release was defined as the absorbance at 490nm minus the absorbance at 600nm, and the LDH release percentage was calculated as experimental LDH release/maximum LDH release × 100%.

**Scanning electron microscope analysis**

BV2 Cells were seeded into 24-well plates with cell climbing slices. When cells had reached 50% confluency, they received different treatments, and then the cell climbing slices were taken out and fixed in 2.5% glutaraldehyde at 4°C for 90 minutes, post-fixed in 1% osmic acid for 20 minutes, dehydrated in ethanol for 10 minutes, gold sputter coated and finally filmed in the scanning electron microscope (SEM) (JEOL JSM-5600LV).

**Statistical procedures**

Graphpad Prism 8.0.1 and SPSS 21.0 were used to carry out the statistical analysis. Kaplan-Meier curves were used to compare the effects of Lira administration on the unexpected death of mice, and Log-rank test was used to compare among different curves and generate hazard ratio. Shapiro-Wilk test was performed to examine whether the quantitative data accord with normal distribution, and analysis of variance (ANOVA) or Kruskal-Wallis test was used to compare quantitative data among more than 2 groups, followed by LSD test or Mann-Whitney test for post hoc comparisons. Independent t test or Mann-Whitney test was utilised to compare quantitative data within 2 groups.

**REFERENCES**

1. FDA U. Estimating the maximum safe starting dose in initial clinical trials for therapeutics in adult healthy volunteers. *Food and Drug Administration Center for Drug Evaluation and Research, US Department of Health and Human Services* [*https://www*](https://www) *fda gov/media/72309/download*. 2005;

2. Wang X, Li B, Liu L, Zhang L, Ma T, Guo L. Nicotinamide adenine dinucleotide treatment alleviates the symptoms of experimental autoimmune encephalomyelitis by activating autophagy and inhibiting the NLRP3 inflammasome. *Int Immunopharmacol*. 2021;90:107092. doi:10.1016/j.intimp.2020.107092
