# Supplementary material for: Liraglutide attenuate central nervous inflammation and demyelination through AMPK and pyroptosis‐related NLRP3 pathway
Source: CNS Neurosci Ther. 2022 Jan 5;28(3):422–34. doi: 10.1111/cns.13791 (PMC8841291; doi:10.1111/cns.13791)
Supplement: Supplementary file 4 — Tab S1‐S2 [file CNS-28-422-s003.doc]

**TABLE S1 Antibodies used in this research**

| **Name** | **Dilution** | **Cat No.** | **Company** | **Use** | **First/Second Antibody** |
| --- | --- | --- | --- | --- | --- |
| Myelin basic protein (MBP) | 1:1000 | GB12226 | Servicebio, Wuhan, China | IF | First |
| Goat Anti-Mouse IgG (H+L) Fluor488-conjugated antibody | 1:1000 | S0017 | Affbiotech, Liyang, China | IF | Second |
| Ionized calcium-binding adapter molecule 1 (Iba-1) | 1:500 | GB12105 | Servicebio, Wuhan, China | IHC | First |
| Phosphorylated adenosine monophosphate activated protein kinase (pAMPK) | 1:1000 | 2535S | CST, Danvers, USA | WB | First |
| Caspase 1 | 1:1000 | Ab179515 | Abcam, Cambridge, UK | WB | First |
| Interleukin 18 (IL-18) | 1:1000 | 57058 | CST, Danvers, USA | WB | First |
| P62 | 1:1000 | 18420-1-AP | Proteintech, Wuhan, China | WB | First |
| Beclin1 | 1:1000 | 11306-1-AP | Proteintech, Wuhan, China | WB | First |
| Microtubule-associated protein 1 light chain 3 (LC3α/β) | 1:1000 | WL01506 | Wanlei, Shenyang, China | WB | First |
| β-actin (ACTB) | 1:50000 | AC004 | Abclonal, Wuhan, China | WB | First |
| Dylight 800 conjugated Goat Anti Rabbit IgG | 1:10000 | A23920 | Abkkine, Wuhan, China | WB | Second |
| Dylight 680 conjugated Goat Anti Mouse IgG | 1:10000 | A23710 | Abkkine, Wuhan, China | WB | Second |

**TABLE S2** Primers used in RT-qPCR

| **Target gene** | **Forward (from 5’ to 3’)** | **Reverse (from 5’ to 3’)** |
| --- | --- | --- |
| Interleukin-1β (IL-1β) | TCAAATCTCGCAGCAGCACATC | CGTCACACACCAGCAGGTTATC |
| Interleukin-6 (IL-6) | TTCTTGGGACTGATGCTGGTG | GCCATTGCACAACTCTTTTCTC |
| Tumor necrosis factor-α (TNF-α) | ATGTCTCAGCCTCTTCTCATTCCT | GGGTCTGGGCCATAGAACTGA |
| RAR-related orphan receptor gamma (RORγt) | GGATGAGATTGCCCTCTACACG | GCGGCTTGGACCACGATG |
| Transforming growth factor-β (TGFβ) | CAACAATTCCTGGCGTTACCT | GCCCTGTATTCCGTCTCCTT |
| T-box 21 (Tbet) | CTTTGAGTCCATGTACGCATCTGT | GGGAACAGGATACTGGTTGGAT |
| Glucagon-like peptide 1 receptor (GLP-1R) | TCTCCAAACTGAAGGCTAATC | CCATCACAAAGGCAAAG |
| Sirtuin 1 (SIRT1) | CGAGGTCCATATACTTTTGTTCAG | GCGTCATATCATCCAGCTCAG |
| P62 | CACTACCGCGATGAGGATGG | CTGCACTTATAGCGAGTTCCCAC |
| LC3 | CCGTCCGAGAAGACCTTCAA | TCTTGCGGCAGGAGAACCTA |
| Beclin1 | CCGTACAGGATGGACGTGGA | TGGGTTTTGATGGAATAGGAGC |
| NLR Family, Pyrin Domain Containing Protein 3 (NLRP3) | TAAGAACTGTCATAGGGTCAAAACG | GTCTGGAAGAACAGGCAACATG |
| PYD and CARD domain containing (ASC) | ACTATCTGGAGTCGTATGGCTTGG | TTCTGTGACCCTGGCAATGAG |
| Caspase1 | GGCTGACAAGATCCTGAGGG | TAGGTCCCGTGCCTTGTCC |
| Gasdermin D (GSDMD) | AGTGCTCCAGAACCAGAACCG | TCACCACAAACAGGTCATCCC |
| IL-18 | AGACCTGGAATCAGACAACTTTGG | GGGTCACAGCCAGTCCTCTT |
| ACTB | GTGACGTTGACATCCGTAAAGA | GTAACAGTCCGCCTAGAAGCAC |
