# Supplementary material for: Liraglutide attenuate central nervous inflammation and demyelination through AMPK and pyroptosis‐related NLRP3 pathway
Source: CNS Neurosci Ther. 2022 Jan 5;28(3):422–34. doi: 10.1111/cns.13791 (PMC8841291; doi:10.1111/cns.13791)
Supplement: Supplementary file 5 — App S2 [file CNS-28-422-s002.docx]

**Appendix S2**

**Experimental autoimmune encephalitis caused significant body weight loss, while Liraglutide administration caused significant extra body weight loss at some timepoints**

In total, 6 batches of experiments were performed (see Table S3 and Table S4 for details) to find liraglutide (Lira) optimal therapeutic dosage for experimental autoimmune encephalitis (EAE) mice. The initial mice dosage (125μg/kg.d, 250μg/kg.d and 375μg/kg.d in batch 1) were converted from clinically approved human dosage for its hypoglycemic roles (0.6mg/d, 1.2mg/d and 1.8mg/d) using equations Food and Drug Administration recommended (see Appendix S1 for detail). Thus, 125μg/kg.d was defined as the minimum mice equivalent dosage (MMED) for human hypoglycemic effect (0.6mg/d). Lira was initially administered subcutaneously (i.c.) daily (qd) or one time two days (qod) after 0 days post immunisation (dpi). However, because unexpected deaths were observed in Lira treated EAE mice (see below), the mice dosage were reduced, and the timepoint as well as frequency of the administration was delayed in batch 3-6.

Compared to healthy control (Ctrl) group, EAE group exhibited significant body weight loss (Figure S1A,B,C). Moreover, Lira enhanced the body weight lowering effect significantly at certain timepoints (Figure S1A,B). However, 1/10 MMED i.c. qod after 8 dpi did not consistently affect the body weight in batch 5 (Figure S1C), as this dosage only caused significant extra weight loss on 10-11 dpi, but on 14, 18, and 21 dpi, the EAE+Lira group had larger body weights than the EAE group.

**Lira administration (>40μg/kg.d) caused unexpected death in EAE mice**

In batch 1-4, a large proportion of Lira-administered EAE mice unexpectedly died before typical disease onset (tail weakness or limb paralysis). After Lira administration, some EAE mice appeared to manifest slow activity and flagging spirit, then developed into sudden death or died without any early signs. In batch 1 (Figure S2A), all mice in the 2-3 MMED group died within 11 days observation window, and their survival curve was significantly worse than the EAE group (*p* < 0.01). Similarly, 37.5% mice of MMED group died.

As an attempt to reduce unexpected death in the following experiment, the dosage, frequency, and administration starting time of Lira were reduced or delayed. In batch 2 (Table S4), the administration starting time was delayed (MMED i.c. qod or qd starting from 4 dpi), and 3/7 mice died unexpectedly within 17 days. Subsequently, in batch 3 and 4 (Figure S2B), the dosage was reduced to 1/2 MMED (i.c. qd starting from 4 dpi), and all Lira administered EAE mice (EAE+Lira group) died unexpectedly within 13 dpi, with the survival curve significantly worse than the EAE group (*p* < 0.001). However, the Lira-administered healthy control mice (Ctrl+Lira group) did not exhibit any unexpected death, suggesting EAE in combination with Lira administration should be the death reason. Next, in batch 5 (Figure S2C), initially, the frequency of administration was reduced (MMED i.c. qod starting from 4 dpi), but owing to a high incidence of unexpected death in this group, the dosage was then reduced to 1/10 of MMED (10μg/kg.d) i.c. qod after 8 dpi, and the survival curve of this group was significantly worse than the EAE group (*p* = 0.015). However, in this batch, the group of mice only received 1/10 MMED i.c. qod starting from 8 dpi did not exhibit unexpected death and showed improved disease scores (Figure 1). To test the safety dosage between 10 and 125 μg/kg.d, 10, 20, 40, and 60 μg/kg.d Lira i.c. qod starting from 8 dpi were tested in batch 6 (n = 3 for every group), however in 40μg/kg.d i.c. qod group, 3/3 mice unexpectedly died (Table S4). In this regard, the dosage between 10-20μg/kg.d qod might be safe. Regarding animal welfare, more experiments were not performed to test the 20μg/kg.d i.c. qod dosage. Taken together, the 10μg/kg.d Lira i.c. qod starting from 8 dpi dosage was utilized to test whether Lira had a neuroprotective effect on mice EAE model.In terms of the reasons for the unexpected death, at first, hypoglycemia was speculated, and blood samples from tail vein were luckily obtained from several moribund mice before the unexpected death (n = 4), then blood glucose level was tested, with the values < 1.1mmol/l, 2.2mmol/l, 5.4mmol/l, and 6.1mmol/l separately. Moreover, we tested several moribund EAE mice after paralysis without Lira intervention, and the blood glucose level was also sometimes low (2.0mmol/l). Thus, hypoglycemia might not be the only cause of death. Some autopsies of unexpected dead mice were carried out, and several mice manifested exuberant secretion of digestive fluids but few food residues in the gastrointestinal tract (data not shown). Still, certain reasons for unexpected death after Lira administration on EAE mice were unknown.

**TABLE S3** Detailed description of 6 batches of EAE mice

| **Batch** | **Date of induction** | **Age (week)** | **Gender** | **Species** | **Body weight (g)** | **Company name** | **Ptx/mice (twice)** | **MOG**  **/mice** |
| --- | --- | --- | --- | --- | --- | --- | --- | --- |
| **1** | 2020.05 | 8-10 | ♀ | C57BL/6N | 16-20 | Nanjing Junke Bioengineering Co., Ltd. | 500ng | 250μg |
| **2** | 2020.07 | 8-10 | ♀ | C57BL/6J | 18-22 | Nanjing Junke Bioengineering Co., Ltd. | 500ng | 250μg |
| **3** | 2020.08 | 8-10 | ♀ | C57BL/6J | 18-22 | SPF (Beijing) Biotechnology Co., Ltd. | 500ng | 250μg |
| **4** | 2020.08 | 8-10 | ♀ | C57BL/6J | 18-22 | SPF (Beijing) Biotechnology Co., Ltd. | 500ng | 250μg |
| **5** | 2020.10 | 8-10 | ♀ | C57BL/6N | 18-22 | Beijing Vital River Laboratory Animal Technology Co., Ltd. | 400ng | 200μg |
| **6** | 2020.12 | 8-10 | ♀ | C57BL/6N | 18-22 | Beijing Vital River Laboratory Animal Technology Co., Ltd. | 500ng | 250μg |

**TABLE S4** Liraglutide dosage and unexpected death of different EAE mice batches

| **Batch** | **Ctrl Number** | **Positive Ctrl (EAE) number** | **Liraglutide dosage (μg /kg.d)** | **Starting date (dpi)** | **Frequency** | **Delivery route** | **Unexpected death** |
| --- | --- | --- | --- | --- | --- | --- | --- |
| **1** | 10 | 8 | 125 (n = 8), 250 (n = 6) and 375 (n = 8) | 0 | qd or qod | i.c. | Yes (Details in Figure S2). |
| **2** | 6 | 2 | 125 (n = 7) | 4 | qd or qod | i.c. | 3/7 mice died unexpectedly within 17 days. |
| **3** | 4 | 5 | 62.5 (n = 10 for EAE and n =3 for Ctrl) | 4 | qd | i.c. | Yes (Details in Figure S2). |
| **4** | 2 | 14 | 62.5 (n = 8 for EAE and n =2 for Ctrl) | 4 | qd | i.c. | Yes (Details in Figure S2). |
| **5** | 10 | 10 | 125 (converted to 10 from 8 dpi, n = 25) | 4 | qod | i.c. | Yes (Details in Figure S2). |
|  |  |  | 10 (n = 10) | 8 |  |  |  |
| **6** | 4 | 18 | 10,20,40 and 60 (n = 3 respectively) | 8 | qod | i.c. | In the 40 μg /kg.d group, 3/3 mice died unexpectedly within 16 days. |
